# Evaluation of acquisition and retention of non-technical skills of residents submitted to interprofessional simulation-based training in pediatric cardiopulmonary resuscitation

**DOI:** 10.1016/j.jped.2024.12.003

**Published:** 2025-02-26

**Authors:** Rafaella Fadel Friedlaender, Eduardo Maranhão Gubert, Cláudia Maria Baroni Fernandes, Rosiane Guetter Mello, Izabel Cristina Meister Martins Coelho

**Affiliations:** a Hospital Infantil Pequeno Príncipe (HPP), Curitiba, Paraná, Brazil; b Faculdades Pequeno Príncipe (FPP), Curitiba, Paraná, Brazil; c Instituto de Pesquisa Pelé Pequeno Príncipe (IPPPP), Curitiba, Paraná, Brazil

**Keywords:** Clinical simulation, Non-technical skills, Skills acquisition, Skills retention

## Abstract

**Objective:**

The purpose of the study was to evaluate the acquisition and retention of non-technical skills by pediatric residents who participated in an interprofessional simulated pediatric cardiopulmonary resuscitation scenario through simulation-based training.

**Method:**

This prospective cohort study was conducted at a simulation center of a Pediatric Hospital. Ninety-six residents of pediatrics and nursing were divided into 16 interprofessional teams and participated in a cardiopulmonary resuscitation simulated scenario followed by a debriefing session. It was conducted twice on the same day and repeated after a period of time that ranged from 107 to 161 days. Groups were evaluated for the acquisition and retention of non-technical skills and global non-technical performance through a valid and reliable tool for measuring teamwork in medical emergencies.

**Results:**

Participants demonstrated an improvement in leadership, teamwork, task management, and overall performance of the team after the first intervention. However, when evaluated during the second intervention, retention of leadership and teamwork were noted, but not for task management and overall performance.

**Conclusion:**

Learning non-technical skills is complex and requires training, ideally with short periodicity, since it demands frequent practice for its acquisition and retention. The present research showed that non-technical skills can be acquired through simulation-based training. However, it was noted that the retention of these skills is more complex, requiring repeated simulations over a longer period of time. Therefore, further research on the learning curve, time to acquisition, and retention of non-technical skills trained with simulation-based education is warranted.

## Introduction

Emergency situations require great skill from healthcare professionals, as they demand quick actions and decisions from the team. In this scenario, providing competent and safe assistance to pediatric patients in Cardiopulmonary Arrest (CA) demands trained professionals focused on compliance with protocols, development of technical and non-technical skills, and frequent training.^1^, ^2^, ^3^

Non-technical skills are defined as cognitive, social, and personal resources that complement technical skills and contribute to the safe and efficient performance of tasks.^4^^,^^5^ These skills include teamwork, leadership, task delegation, communication skills, situational awareness, task management, stress management, professionalism, clinical reasoning, decision-making, among others.^1^

Teaching non-technical skills is fundamental and has a decisive impact on the quality and the outcome of patient care.^4^ Repeated training associated with feedback is a key factor in gaining and improving this competence.^6^ Therefore, they should be valued in health professionals' teaching and learning processes.^1^

Clinical simulation is proven to be effective in training these skills and has been one of the most used methods for their development, through training with multidisciplinary teams, promoting the development of high-performance teams, resulting in improvements in the quality of care and patient safety and, consequently, in the final clinical outcome.^1^^,^^4^^,^^7^

An important question when evaluating skills training is their long-term acquisition and retention and what is the best way to teach them. The concept of learning is related to the ability to understand new information or knowledge instructed by an intervention. Retention refers to the ability to maintain what has been learned over time.^8^

It is known that skill retention is a challenge. However, until now, no study has proven the length of time necessary to carry out new training for long-term retention of these skills, as well as what is the best teaching method for those.^2^

Therefore, this study aimed to evaluate the acquisition of non-technical skills by pediatric nursing and medicine residents as well as the retention after a new approach, through a pediatric CA scenario simulation. The hypothesis is that, after day 1, there would be an improvement in all variables assessed regarding non-technical skills.

## Methods

This study consists of a prospective cohort to evaluate an educational intervention. Participants were pediatric nursing and medicine residents at the institution. All 96 members of the residency program were invited to participate. Of these, 43 were first (*n* = 23) and second-year (*n* = 20) nursing residents and 53 were first (*n* = 21), second (*n* = 16), and third-year (*n* = 16) pediatric residents. Among the 96 residents, 85 agreed to participate in the research. Considering 96 residents enrolled in residency and a sample size of 85 participants, the margin of sampling error was 3.6 % (96.4 % confidence interval), which is below the standard 5 % error (95 % confidence interval).

Residents participated in a simulated pediatric CA scenario followed by a debriefing session at three different moments: Moment 0 (M0) – first pediatric CA simulation followed by debriefing; Moment 1 (M1) – second simulation followed by debriefing; and Moment 2 (M2) – third simulation followed by debriefing. M0 and M1 were carried out on the same day, in April and May of 2022. M2 occurred after a time interval that ranged from 107 to 161 days in relation to M0 and M1, in August and September of 2022, in the Simulation Center of a reference Pediatric Hospital in Curitiba-PR, Brazil.

Residents were divided into 16 teams of four to seven members, consisting of at least one resident from each year of pediatrics (except for one group that did not have one from the second year) and at least one nursing resident from each year (except for three groups that did not contain one from the second year). Some groups contained more than one resident from the same year. On the first day, the sample consisted of 85 members, including 20 first-year and 13 second-year nursing residents, totaling 33 nurses and 21 first-year, 15 second-year, and 16 third-year medical residents, totalizing 52 medical participants. On the second day, 58 individuals attended the study: 14 first-year and four second-year nursing residents, totalizing 18 nursing participants, and 17 first-year, 11 second-year, and 12 third-year medical residents, totalizing 40 medical residents of pediatrics. Therefore, in M0 and M1 there were 16 teams, while on the second day, 12 groups.

The first session (M0 and M1) lasted 2 h and the second (M2) lasted 1 h. The simulated scenario lasted 10 min and was the same in all three moments and was a clinical scenario of a 6-year-old male patient who developed a CA in ventricular fibrillation (VF). The choice of the rhythm in VF, despite not being the most common in the pediatric population, was purposefully chosen to increase the degree of technical and non-technical skill difficulty. A situation of high complexity and less frequency in daily clinical practice demanded more from the team in terms of leadership, communication, task management, and effective teamwork.

Participants received guidance on the simulation laboratory, on the operation and limitations of the simulators, material, and equipment available in the room, as well as on the scenario in which they participated. All teams maintained the same participants in all simulated events. Before each simulation, residents decided who would be the leader and other team roles. The leader chosen was the medical resident of a higher degree and all roles were maintained through all scenarios. Participants were not used to participating in frequent simulation training and some of them used to work together at clinical service, but not on the same team on a regular basis.

All three instructors who carried out the simulation and debriefing session have a pediatric board certificate and received previous training in simulation. They used the same structured model for the debriefing – called GAS (Gather, Analyze, and Summarize) – which is composed of three phases. The first (Gather) brings together the events experienced during the simulation, the second (Analyze) promotes reflection by participants and the third (Summarize) reviews the key points of the discussions. GAS is a validated, structured and supported debriefing method that was designed to standardize the debriefing for the American Heart Association courses: Advanced Cardiac Life Support (ACLS) and Pediatric Advanced Life Support (PALS). It is also one of the framework methods recommended by the International Nursing Association for Clinical Simulation and Learning (INACSL) in their Standards of Best Practice for Simulation.^9^, ^10^, ^11^

Non-technical skills – teamwork, leadership, and task management – and the overall assessment at all times were evaluated and compared. The research instrument used was the TEAMTM tool (Team Emergency Assessment Measurement) questionnaire – which is a validated tool, built to evaluate teamwork in medical emergencies. It consists of twelve items, the first eleven evaluating the team's performance, using a Likert scale from 0 to 4 points in the following categories: leadership, teamwork, and task management. Question number twelve, which corresponds to the team's global non-technical performance, is scored on a scale of 0 to 10.^12^^,^^13^

This instrument was chosen among the multiple options because of its previous validation and translation into Portuguese, its easy application, its assessment of the group performance as a whole, and because it considers important non-technical skills, such as leadership, task management, and teamwork, in addition to including an item on the global evaluation of the non-technical skills performance. Also, it is available on its own website freely and in several other languages. All authors received training on how to interpret the tool and they were present during all simulated scenarios and answers were discussed before deciding the final score for each item of the questionnaire.

Data were analyzed using statistics, calculating proportions in percentages, and were presented in tables created in the Microsoft Excel 2007 program. The statistical program used was the R Version 4.0.2 model (R Core Team, 2020). The descriptive analysis was carried out by checking quantities and percentages for categorical variables and descriptive measures (minimum, maximum, quartiles, mean, and standard deviation) for continuous variables. Student's T-test was applied and a *p*-value < 0.05 was considered for statistical significance.

### Ethics approval statement

The study was approved by the Ethics and Research Council of the Faculdades Pequeno Príncipe (approval number of the research was 5.131.695). All enrolled participants signed the informed consent and image use forms before the study and were informed regarding the phases of the research.

## Results

Of all residents, 85 participants were included, 33 of whom were first and second-year nursing residents and 52 first, second, and third-year pediatric residents, who were divided into 16 teams at M0 and M1. At M2, 58 residents participated in the scenario, totalizing 12 groups for analysis (4 groups were excluded in M2 because groups were incomplete, which would cause bias and hinder the analysis of skill retention).

Among all participants, 84.7 % were female, with an average age of 26 years (ranging from 22 to 36 years) and their training time from 2 to 89 months (an average of 19 months). Half of the first-year nursing resident group participated in a basic life support course in pediatrics before the first intervention, but none of the second-year nursing residents took a course related to pediatric CA before participating in the research. Among medical residents, 42 % had taken a pediatric emergency course before the intervention and all participated in a class on pediatric CA one month before the first simulation.

The means and standard deviation (SD) of the responses obtained in M0 and M1 and in M1 and M2 were compared, and divided into the following categories: leadership (questions 1 and 2), teamwork (questions 3 to 9), task management (questions 10 and 11) and global assessment (question 12).

It is observed that in M0 and M1, there is a significant difference between all dimensions evaluated (Table 1). When comparing the average responses between M1 and M2, there was a significant difference between leadership and teamwork, while there was no statistical relevance between the variables of task management and global assessment (questions 10 and 11) (Table 2).

**Table 1:** Comparison of response means between M0 and M1.

Category	M0	SD	M1	SD	*p*
Leadership	1.28	0.99	2.44	1.24	< 0.001
Teamwork	1.88	0.85	2.77	0.9	< 0.001
Task Management	2.09	0.89	3	0.8	< 0.001
Global	5.12	1.5	7.62	1.36	< 0.001

SD, Standard deviaton.

**Table 2:** Comparison of response means between M1 and M2.

Category	M1	SD	M2	SD	*p*
Leadership	2.44	1.24	3.29	0.75	< 0.05
Teamwork	2.77	0.9	3.19	0.75	< 0.001
Task Management	3	0.8	2.96	0.62	0.83
Global	7.62	1.36	8	0.95	0.4

SD, Standard deviaton.

The mean result obtained in question 12 was compared at different moments and there was statistical significance between M0 and M1, while there was no statistical difference when comparing the values found at M1 with M2 (Table 3).

**Table 3:** Global assessment comparison between M0 and M1 and M1 and M2.

	M0	SD	M1	SD	*p*
Question 12	5.12	1.5	7.62	1.36	< 0.001
	M1	SD	M2	SD	*p*
Question 12	7.62	1.36	8	0.95	0.4

SD, Standard deviaton.

## Discussion

This study evaluated the acquisition and retention of non-technical skills –leadership, teamwork and task management, as well as the global assessment scale of the team's performance as a whole – of pediatric medicine and nursing residents after a pediatric CA simulated scenario followed by a debriefing session in a Simulation Center.

The means and standard deviation of the questionnaire responses were calculated according to four variables and compared between M0 and M1 (acquisition) and M1 and M2 (retention). In line with data from the literature, it is noted that after the first intervention, when observing the immediate acquisition, the main result is that all 16 groups have improved in all variables analyzed, which demonstrates the effectiveness of simulation and debriefing in the acquisition of these skills.^4^

According to Brandão et al. and Rey et al., simulation is proven to be effective for teaching complex procedures as it allows the reproduction of real-life scenarios in a safe environment, without exposing patient safety to risk. Furthermore, it is also a resource for working on important non-technical components in professional training, such as resource management in critical events, teamwork, relationships between teams, leadership, and effective communication.^1^^,^^14^

The present study demonstrated that when comparing the mean responses in M1 and M2, leadership and teamwork variables were statistically relevant, demonstrating that there was retention of these skills. However, task management and global assessment did not obtain a statistically significant difference, which demonstrates that there was no retention of these skills after the second approach.

Evidence suggests that, associated with technical skills, human factors such as teamwork and leadership affect adherence to CA algorithms and protocols and, consequently, patients' clinical outcomes.^15^ This fact was demonstrated in this study, as shown by questions about task management (question number 10 – which assesses whether the team prioritized tasks and question 11, referring to adherence to approved protocols and guidelines) which did not demonstrate statistical relevance when comparing M1 and M2. The fact that there was no improvement in this item may justify the lack of improvement in the team's general performance in the second moment.

Simulation is known to be an effective methodology for teaching potentially serious and rare events. However, few studies assess the time required for non-technical skills to be acquired, as well as their retention time. Furthermore, few studies evaluate the complexity of teaching these skills to a population of novice students. Moreover, the learning curve for non-technical skills is variable and complex, which requires regular training, especially in high-complexity, low-frequency clinical situations.^16^

Guerreiro et al. aimed to assess the behavioral skills of 12 second-year fellows in neonatology before and after a simulation training program on neonatal resuscitation, with three training cycles of 1 month followed by a 3-month interval. The results showed that their overall behavioral performance and specific skills (communication, delegation of tasks, allocation of attention, use of information and resources) improved after the second month of training.^17^

In the present study, residents participated in three interprofessional simulated scenarios, which may not have been sufficient to retain these skills. Furthermore, they are beginner learners, which may require more training sessions and more time to learn these skills. Some studies demonstrate that the time for the decline of these skills is uncertain and may vary according to the specific skill, the level of learning and the time between its teaching and assessment.^16^ Other studies show that deterioration of skills is seen shortly after training and some authors suggest that knowledge is generally better retained.^18^^,^^19^

Research done with pediatric residents demonstrated that neonatal resuscitation skills decline with time after completion of a neonatal resuscitation program (NRP) course. The skill could be maintained after a 2-month interval but not after 4 months or 1 year after training. Residents are particularly at risk for skill decay because training programs have different rotations and, also, expert practitioners would probably not experience such forgetting curves given that they regularly practice these habilities.^18^

According to the literature, when leadership is carried out clearly, there is greater cooperation between team members as well as improved task execution. Furthermore, successful teams demonstrate significantly more leadership behavior, clearer task delegation, a tendency to greater and better information transfer, and fewer conflicts between interprofessional team members.^15^ Residents are expected to have less experience in dealing with emergency situations, especially regarding leadership, as it is a complex skill that requires continued training and experience in this clinical context. In this study, the improvement of this skill may have contributed to the improvement of the interprofessional teams' performance in general, in all the evaluated aspects.

In a randomized controlled study, using a simulated cardiac arrest scenario, it was evaluated whether leadership teaching translated into greater leadership initiative by the team, as well as improved CPR performance. Participants were medical students, who watched a simulation on cardiac arrest and were subsequently divided into two groups: one received instructions focused on technical skills, such as correct hand positioning during cardiac resuscitation, while the second was taught non-technical skills, such as leadership and communication. After four months, a simulation session was carried out comparing both groups. The result was that the group that had received guidance on leadership demonstrated less interruption time in cardiac resuscitation, faster initiation of CPR maneuvers, and greater leadership initiative. The group with technical instructions demonstrated a greater ability to perform cardiac massage correctly, assessed through the correct positioning of the hands and shoulders.^20^

This study had some limitations: some participants were absent, which did not allow complete analysis of the data for all groups at M2. This may have interfered with the mean of the results on the retention of the assessed skills. Furthermore, the sample is made up of residents, who probably have less experience with critical situations, which may have affected the result of the analysis. Another important issue is the fact that learning these skills does not guarantee that the resident will apply them in their daily practice, since in a simulation laboratory they may act differently because they are being observed. Moreover, emergency care - such as CA - is stressful and much more difficult in real life, which can alter the performance of each member and the group as a whole when inserted in their workplace.

Technical and non-technical skills are closely interconnected and their performance by the professional directly affects the safety and quality of patient care, as well as the outcome. Teaching and, mainly, learning non-technical skills – such as leadership, teamwork, effective communication, and conflict management – is complex and requires frequent training, ideally on a short basis, as these are skills that require frequent practice to be acquired and retained.^15^^,^^16^

In conclusion, this research demonstrates that non-technical skills can be acquired after training through clinical simulation, as there was statistical significance in all variables analyzed (leadership, communication, teamwork, task management, and global performance assessment). However, when evaluated after a new approach, retention of leadership and teamwork were noted, but not for task management and overall performance, which implies the need to carry out training on a shorter basis and greater frequency, especially for participants with less experience who are still in the training process.

This study highlights that clinical simulation is a proven effective methodology for training these skills. So, future training programs should focus on practices to improve non-technical skills, preferably in a multi-professional way, since this is the situation that professionals will face in their real clinical context, as they interfere with patient care and patient final clinical outcome, as well as the psychological safety of the team as a whole.

This research acts as an incentive to carry out new research in seeking answers regarding the learning curve and the time required to acquire non-technical skills. Likewise, the duration of their retention, to define effective teaching methods, as well as the necessary periodicity for their training.

## Conflicts of interest

The authors declare no conflicts of interest.
